# Subvalvar Mitral Aneurysm: A Rare Cause of Mitral Leak

**DOI:** 10.14740/cr408w

**Published:** 2015-06-11

**Authors:** Santosh Kumar Sinha, Chandra Mohan Verma, Ramesh Thakur, Varun Kumar, Mohit Sachan, Ashutosh Kumar, Mukesh Jitendra Jha, Vikas Mishra

**Affiliations:** a Department of Cardiology, LPS Institute of Cardiology, G.S.V.M. Medical College, G. T. Road, Kanpur, Uttar Pradesh 208002, India

**Keywords:** Arrhythmias, Mitral regurgitation, Palpitation, Submitral aneurysm

## Abstract

Submitral aneurysm (SMA) results from outpouching of the left ventricular wall which is congenital and occurs adjacent to the posterior leaflet of the mitral valve. Although it is predominantly described among the natives of Africa, it is considered rare in the Indian subcontinent. It presents clinically as palpitation, congestive cardiac failure in the presence of mitral regurgitation, arrhythmias and embolic phenomenon. Echocardiography is the precise diagnostic tool. We report this case of a 24-year-old man who was referred for evaluation of palpitation. This underlines the importance of considering SMA in the differential diagnosis of mitral regurgitation in young patients in our subcontinent where rheumatic etiology usually predominates.

## Introduction

Subvalvar aneurysm (SMA) is a rare cardiac anomaly. It has been described predominantly in the African populations, though it has been sparingly reported in mixed races and Caucasians and very few cases have been reported from India [1]. Though multiple etiologies have been proposed for the development of this condition, the current opinion is that SMAs are most likely due to a congenital weakness of the fibrous annulus of the valve which leads to outpouching of left ventricular wall which invariably occurs adjacent to the posterior leaflet of mitral valve [2-4]. It has been postulated that a dehiscence of the fibro-muscular union will result in aneurysm formation [5]. As large part of the mitral annulus is related to the posterior leaflet which is attached to the myocardium of the left ventricle by annular ring, the immediate external relationship of the mitral ring is the epicardium of the atrioventricular groove. As dehiscence of this union results in occurrence of the SMA, it will be invariably below the mitral leaflet [5].

## Case Report

**Figure 1:**
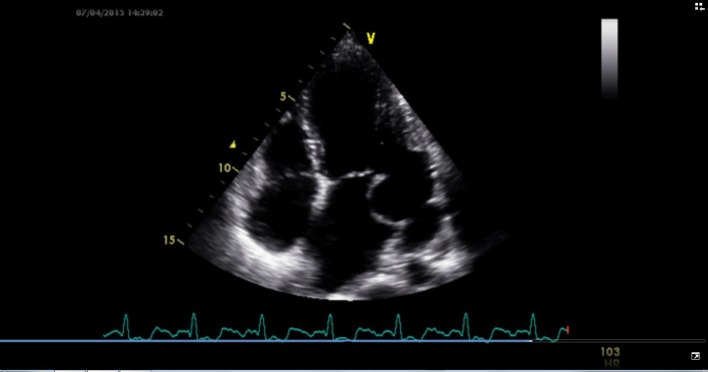
Transthoracic echocardiography in apical four-chamber view showing submitral aneurysm in the posterolateral wall of the left ventricle.

**Figure 2:**
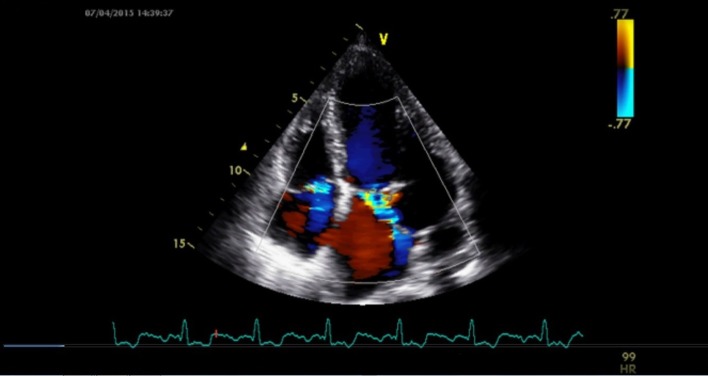
Color Doppler showed severe MR with an eccentric jet with severe tricuspid regurgitation.

**Figure 3:**
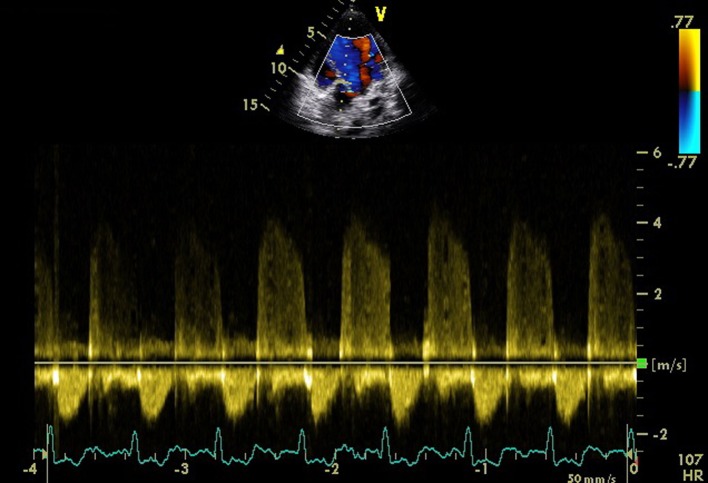
Color Doppler in apical five-chamber view showed mild aortic regurgitation.

**Figure 4:**
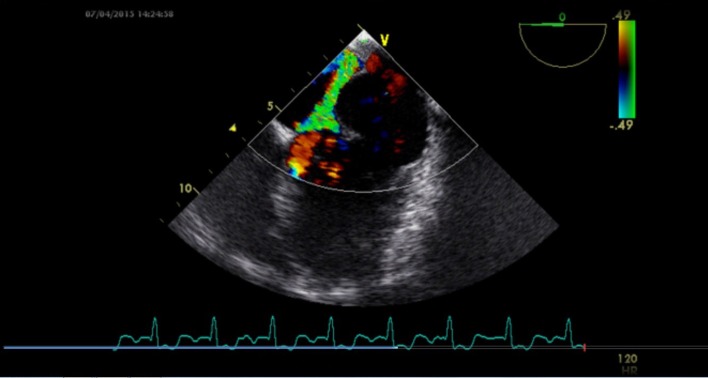
Transesophageal echo in four-chamber view with transducer positioned in esophagus displaying severe mitral regurgitation with an eccentric jet.

A 24-year-old male presented for evaluation of palpitations. There was no history suggestive of rheumatic fever. On examination, blood pressure was 104/60 mm Hg in right upper limb in supine position and pulse rate was of 86/min. Jugular venous pressure was normal in height with C-V waveform. The apex was in the sixth left ICS, 2 cm outside the mid-clavicular line and hyperkinetic in character. On auscultation, S1-soft, S2-wide variable split with loud P2 and LVS3 were present. A grade 4/6 pan-systolic murmur radiating towards the axilla was heard. There was also a pan-systolic murmur in the left third parasternal region which increased during inspiration. ECG showed normal sinus rhythm. Chest X-ray revealed cardiomegaly with evidence of pulmonary venous congestion. On transthoracic echocardiography in apical four-chamber view, it showed SMA (Fig. 1) in the postero-lateral wall of the left ventricle. Color Doppler showed severe mitral regurgitation with an eccentric jet towards left atrium and severe tricuspid regurgitation (Fig. 2). Color Doppler in apical five-chamber view showed mild aortic regurgitation (Fig. 3). Transesophageal echo in four-chamber view with transducer positioned in esophagus displayed severe mitral regurgitation with an eccentric jet (Fig. 4). So, SMA was diagnosed and referred to cardiothoracic department for further management.

## Discussion

SMA is a rare entity that occurs most often in the black population. It was first described in 1812 by Corvisart. The prevalence of this lesion among blacks appears to indicate a congenital origin or predisposition [6, 7]. SMA is probably caused by a junctional defect between the cardiac muscle and the fibrous structure of the heart [4]. Sporadic case reports in the last two decades have documented their existence in the Indian population also though very rare. Although the etiology of the condition is thought to be a congenital, co-existence of the condition with Takayasu’s arteritis and tuberculous pericarditis [8] has been reported. The most common presentation is mitral regurgitation, but it can rarely present as life-threatening complications such as ventricular tachycardia due to compression of the left main coronary artery [8]. Doppler echocardiography is the most valuable tool to diagnose SMAs in the clinical setting. In Indian subcontinent, most common cause of mitral leak in young age group is rheumatic, so SMA, although uncommon, should always be considered as an etiology of mitral leak in young patients. The definitive diagnosis is made by echocardiography and definitive treatment is surgical.
